# Quality and acceptability of patient-reported outcome measures used to assess fatigue in axial spondyloarthritis (axSpA): a systematic review (protocol)

**DOI:** 10.1186/s13643-018-0777-7

**Published:** 2018-08-07

**Authors:** Nathan A. Pearson, Jonathan C. Packham, Helen Parsons, Kirstie L. Haywood

**Affiliations:** 1 Royal College of Nursing Research Institute, Division of Health Sciences, Warwick Medical School, University of Warwick, Coventry, UK; 2 Institute of Applied Clinical Science, Keele University, Staffordshire, UK; 3 Warwick Clinical Trials Unit, Warwick Medical School, University of Warwick, Coventry, UK

**Keywords:** Quality, Acceptability, Fatigue, Ankylosing spondylitis, Axial spondyloarthritis, Measurement, Musculoskeletal disorders, Systematic review

## Abstract

**Background:**

The prevalence of axial spondyloarthritis (axSpA) is estimated between 0.15 and 1.2%, with many of those patients experiencing severe fatigue. Current axSpA assessment guidance recommends use of a single-item visual analogue scale for fatigue severity. However, concerns have been raised about the ability of such a limited assessment to identify patients with major fatigue, to detect important change in fatigue or to reflect the multi-dimensional nature of fatigue. The proposed systematic review will identify and evaluate the quality and acceptability of single- and multi-item patient-reported outcome measures (PROMs) used to assess fatigue in axSpA, seeking to make recommendations for the ‘best’ measures for research and/or clinical practice.

**Methods/design:**

The review will seek to include published studies which report evidence of the development and/or measurement and/or practical properties of clearly defined and reproducible measures of fatigue following completion by patients with axSpA. Five major databases will be searched from 1980 to August 2017: MEDLINE (OVID), EMBASE (OVID), PsycINFO (OVID), World of Science and CINAHL. Study methodological quality will be assessed against the COnsensus-based Standards for the selection of health Measurement INstruments (COSMIN) checklist. The measurement and/or practical properties of reviewed measures will be assessed against current international standards. A short list of the ‘best’-quality PROMs will be produced. The review will be reported in accordance with the Preferred Reporting Items for Systematic Reviews and Meta-Analyses (PRISMA) guidelines.

**Discussion:**

This study will provide the first robust and transparent evaluation of patient-reported measures of fatigue used in the axSpA population, synthesising evidence of quality, relevance and acceptability. The review will benefit patients, clinicians, health professionals and researchers wishing to enhance axSpA-fatigue assessment in routine practice, service evaluation and research. The findings will impact future research which seeks to better understand the nature of axSpA fatigue and evaluate the relative benefit of fatigue-management strategies.

**Systematic review registration:**

PROSPERO CRD42016042271

**Electronic supplementary material:**

The online version of this article (10.1186/s13643-018-0777-7) contains supplementary material, which is available to authorized users.

## Background

Axial spondyloarthritis (axSpA) is an inflammatory disease that primarily affects the spine and pelvis, impairing mobility to the detriment of the patient’s physical wellbeing [1]. It is characterised by widespread back and joint pain and stiffness [2]. axSpA and ankylosing spondylitis (AS) are two facets of a single disease distinguished by whether radiographic sacroiliitis is observable by X-ray examination (AS) or not (axSpA) [2]. Up to 75% of axSpA patients report experiencing fatigue [3–7], and for many, this is both severe and frequent [7]. Patients with AS have highlighted the relative importance of seeking to better understand the fatigue associated with their illness [8], underlining the need for an appropriate fatigue assessment that really captures what matters to patients. As exemplified in rheumatoid arthritis (RA), a review of methods of fatigue assessment [9] and qualitative research with patients highlighted both the multi-faceted nature of RA fatigue [10] and the inadequacy of current methods of assessment. The result was the development of a patient-reported outcome measure (PROM) specific to RA fatigue—the Bristol Rheumatoid Arthritis Fatigue Multidimensional Questionnaire (BRAF-MDQ) [11, 12]. PROMs are single- or multi-item questionnaires which seek to provide a patient-derived assessment of how they feel, what they can and cannot do and how well they are living their lives as a consequence of their health and associated health care [13].

Current assessment guidance for AS exists in the form of a core outcome set (COS) [14]. The COS provides guidance for the minimum number of outcomes to include in future clinical practice and clinical trials: that is, pain, stiffness, function, global wellbeing and spinal mobility [14, 15]. A recent update recommends the assessment of fatigue severity with a single-item visual analogue scale (VAS) [16]. However, evidence from a large UK cohort of AS patients highlighted significant limitations with a single-item assessment of fatigue, including the failure to identify some patients with major fatigue, to detect important change in fatigue, to reflect the multi-dimensional nature of fatigue or to detail the nuances of fatigue essential to driving tailored healthcare.

A growing number of multi-item fatigue-specific (for example, the Multidimensional Fatigue Inventory (MFI-20) [17] and Fatigue Severity Scale [18]) and condition-fatigue-specific (for example, the BRAF-MDQ [12] and Functional Assessment of Cancer Therapy scale [19]) PROMs are now available. This growth in availability reflects the importance of capturing the multi-faceted, often condition-specific, nature of fatigue [20] and the importance of seeking to reflect the outcomes that really matter to patients [21]. However, it is unclear how well these measures perform in the axSpA population; it cannot be assumed that the measurement and practical properties of measures are consistent across different patient populations. Confidence in the use of PROM-based assessment is underpinned by evidence of performance in the population of interest [22], and structured reviews of PROM quality and acceptability provide essential evidence to inform selection.

The proposed systematic review will evaluate the quality (measurement properties), relevance (measures what is important) and acceptability (simplicity and convenience) of clearly defined and reproducible multi- and single-item PROMs which purport to measure fatigue and have been completed by the axSpA population. A short list of ‘best’ measures of fatigue for use with the axSpA population will be developed to inform recommendations for use in both routine clinical practice and research.

## Method/design

The review will include published studies reporting evidence of the development, measurement and/or practical properties of clearly defined and reproducible measures of fatigue evaluated following completion by patients with axSpA. The review will be completed and reported in accordance with the Preferred Reporting Items for Systematic Reviews and Meta-Analyses (PRISMA) guidelines [23].

Study methodological quality will be assessed against the COnsensus-based Standards for the selection of health Measurement INstruments (COSMIN) guidelines [24, 25]. The measurement and/or practical properties of included measures will be assessed against a transparent appraisal framework informed by current international standards for PROM quality [22, 26–29].

### Search strategy

A comprehensive search strategy will be developed using Medical Subject Headings (MeSH) and free text searching to reflect four key characteristics [26, 30, 31]: (1) population—axSpA, (2) construct—fatigue, (3) type of assessment—patient-reported measures and (4) measurement properties (a modified version of a sensitive search filter and accompanying exclusion filter) [30] (example search in Additional file 1: Appendix 1).

Following the review of titles and abstracts of included studies, a further ‘named measure’ search will be developed and applied with search terms developed as above to reflect (1) population—axSpA, (2) construct—fatigue, (3) named fatigue measures and (4) measurement properties.

The search strategy will be modified for each of the following databases: MEDLINE (OVID), EMBASE (OVID), PsycINFO, World of Science and CINAHL. Searches will be run from 1980 to August 2017.

#### Stage 1: Identifying evaluative studies of PROM-based fatigue assessment in axSpA

The search strategy will use MeSH, keywords and synonyms to identify studies of adult patients with axSpA where the concept of fatigue is assessed. To ensure maximal sensitivity, a wide range of terms will be used to reflect the target patient sample (axSpA) and fatigue. A modified filter describing measurement and assessment will be used to identify studies using PROMs (original filter developed by the PROM group and Knowledge Centre, Department of Public Health, University of Oxford). Terms describing the measurement evaluation will be searched for using a modification of the COSMIN filter [30]. The recommended COSMIN exclusion filter will be added to the search string [30].

Titles and abstracts will be reviewed for inclusion by one reviewer (NP); a second reviewer (KH) will independently review a 10% subset of randomly selected titles and abstracts and agreement checked [32, 33]. A third independent reviewer (JP) will resolve any differences regarding eligibility. Reference lists of included articles will be screened for additional articles. The reason(s) for any full-text exclusions will be recorded.

### Review inclusion/exclusion criteria

#### Study inclusion

Studies will be included if they (i) include a clearly identifiable and reproducible PROM-based assessment of fatigue; (ii) the study reports evidence of the development and/or evaluation (practical or measurement properties) of the PROM following completion by members of the axSpA population; and (iii) the study has been published, peer-reviewed, is available as a full text and is written in English. Studies will be excluded if they are (i) available as abstract only; (ii) the assessment of fatigue is not patient-reported, clearly identifiable or reproducible; (iii) the study simply describes use/application of a PROM without further evidence of measurement/practical properties; (iv) the measurement and/or practical properties cannot be extracted specific to the axSpA population; or (v) the study has not been published, peer-reviewed or is not available in English.

All abstracts that include patients with psoriatic arthritis (PsA) will be screened by a consultant rheumatologist (JP) and included if a subset of patients is clinically defined as having axSpA and separately reported.

#### PROM inclusion

PROMs will be included if (i) they are fatigue specific: both multi-item and single-item measures will be included and (ii) fatigue is assessed as a separate domain within a multiple domain assessment (e.g. SF-36 Vitality [34]). Assessments of fatigue will not be included if they are (i) clinician-reported or (ii) a non-PROM-based assessment.

#### Stage 2: Identifying studies using named PROM-based fatigues measures in axSpA

The search strategy will use the search filters developed in stage 1 for population (axSpA), construct (fatigue) and measurement properties (COSMIN filter). In addition, a named measures search filter will be developed and added to the search string to identify single- and multi-item measures used in axSpA fatigue assessment. All identified titles and abstracts will be extracted, and any duplicates between stage 1 and 2 searches will be removed. Title and abstract screening will follow the same procedure outlined for the stage 1 search (see stage 1), and the same eligibility criteria will be applied.

#### Data extraction and appraisal

Data extraction will be informed by previous reviews [35, 36] (see Additional file 1: Appendix 2) and the requirements of the COSMIN checklist [24, 25]. COSMIN provides a transparent appraisal system that is internationally developed. The checklist contains quality criteria for evaluating ten measurement properties—validity (content, structural, construct, criterion, cross-cultural), reliability (internal consistency, test-retest, measurement error), responsiveness and interpretability. This review will consider all evidence of measurement evaluation that relates to fatigue-specific measures only.

Data extraction will capture (1) study information (population, definition of fatigue, language) and (2) PROM-based information. PROM-based evidence of measurement properties will include validity (structural, content and face, construct, criterion, longitudinal), reliability (inter- or intra-rater, test-retest, internal consistency, measurement error), responsiveness (criterion or construct-based) and interpretability (minimal important change, smallest detectable change, response shift). Evidence for the practical properties of PROMs will include acceptability (relevance) and feasibility. The extent of patient involvement as active research partners in PROM development, evaluation and/or application will be sought [37].

Study methodological quality for each reported measurement property will be assessed using the COSMIN checklist 4-point scale (i.e. poor, fair, good, excellent) [24, 25]. Two reviewers (NP and KH) will independently undertake data extraction and apply the COSMIN checklist on a randomly selected 10% subset of included papers. Any disagreements are resolved through discussion with a third reviewer (JP or HP).

#### Data synthesis

Data synthesis will seek to contextualise evidence of the reported measurement and/or practical properties alongside the methodological quality of the study. As per earlier reviews, data synthesis will consider (i) study methodological quality (COSMIN scores), (ii) the number of studies reporting evidence per fatigue measure, (iii) the results for each practical and measurement property per measure, and (iv) consistency between evaluations [35]. Data synthesis will report two pieces of information. First, measurement property quality will be categorised as adequate (+), not adequate (−), conflicting (+/−) or unclear (?). Second, the strength of evidence for the quality of each measurement property reviewed will be categorised as ‘strong’, ‘moderate’, ‘limited’, ‘conflicting’ or ‘unknown’ [32, 35].

Following data synthesis and item-content comparisons, PROM recommendations will be informed by (1) whether and to what extent essential domains of fatigue identified—as per the RA-fatigue model—are reflected within the PROM (content validity), (2) the availability of adequate evidence of minimally important measurement properties—validity (structural and construct) and reliability (internal consistency and test-retest), and (3) an evidence base that is minimally judged to be of moderate quality.

## Discussion

Awareness of the importance of fatigue in inflammatory conditions has grown over the past decade [20]. Whilst patients report fatigue as one of the key symptoms of their condition, there is limited evidence of an improved understanding of fatigue in axSpA and its impact on patients’ lives [3, 38, 39]. Moreover, growing evidence suggests that the experience of fatigue is not homogeneous across conditions [10, 40] and hence a generic approach to assessment may miss important aspects of fatigue for particular patient groups.

This study will provide the first robust and transparent evaluation of patient-reported measures of fatigue used in the axSpA population, synthesising evidence of quality, relevance and acceptability. The findings of this review will inform the selection of patient-reported fatigue assessment, thus impacting future research which seeks to better understand the nature of axSpA fatigue. Improving the assessment of fatigue in routine practice, service evaluation and research will enhance our understanding of the way in which fatigue impacts upon the lives of people with axSpA, and the way in which their fatigue responds to fatigue-management strategies.

## Additional file

Additional file 1: Appendix 1. Search strategy. Appendix 2. Quality criteria to appraise reported measurement properties [28, 36]. (DOCX 25 kb)

## Abbreviations

AS: Ankylosing spondylitis
axSpA: Axial spondyloarthritis
CINAHL: Cumulative Index to Nursing and Allied Health Literature
COS: Core outcome set
COSMIN: COnsensus-based Standards for the selection of health Measurement INstruments
PRISMA: Preferred Reporting Items for Systematic Reviews and Meta-Analyses
PROM: Patient-reported outcome measure
RA: Rheumatoid arthritis
