# Supplementary material for: Quality and acceptability of patient-reported outcome measures used to assess fatigue in axial spondyloarthritis (axSpA): a systematic review (protocol)
Source: Syst Rev. 2018 Aug 7;7:116. doi: 10.1186/s13643-018-0777-7 (PMC6081943; doi:10.1186/s13643-018-0777-7)
Supplement: Supplementary file 1 — Appendix 1. Search strategy. Appendix 2. Quality criteria to appraise reported measurement properties [28, 36]. (DOCX 25 kb) [file 13643_2018_777_MOESM1_ESM.docx]

**Appendix 1: Search Strategy**

Search 1 – PROM evaluation studies

Database: Ovid MEDLINE(R) <1946 to August Week 5 2017>, Embase Classic+Embase <1947 to 2017 September 08>, PsycINFO <1967 to September Week 1 2017>

Search Strategy:

--------------------------------------------------------------------------------

1 Spondylitis, Ankylosing/ or (Axial Spondyl* or Spondyl*, Axial or (Ankylosing Spondyl* or Spondyl*, Ankylosing) or Spondyloarthropath* or Spondyloarthritis).ti,ab. (49090)

2 (Fatigue or Asthenia* or Lassitude or Exhaust* or Inertia or Drows* or Letharg* or (Tiring or Tired* or Weary or Weariness)).mp. (544147)

3 (HR-PRO or HRPRO or HRQL or HRQoL or QL or QoL or (PRO or PROs or PROM or PROMs)).ti,ab. or (VAS or NRS).mp. or visual analogue scale*.mp. or numeric* rating scale*.mp. or quality of life.mp. or (health index* or health indices or health profile*).ti,ab. or health status.mp. or ((patient or self or proxy) adj (appraisal* or appraised or report or reported or reporting or rated or rating* or based or assessed or assessment*)).ti,ab. or ((disability or function or functional or functions or subjective or utility or utilities or wellbeing or well being) adj2 (index or indices or instrument or instruments or measure or measures or questionnaire* or profile or profiles or scale or scales or score or scores or status or survey or surveys)).ti,ab. (2092925)

4 (instrumentation or methods).sh. or (Validation Studies or Comparative Study).pt. or exp Psychometrics/ or psychometr*.ti,ab. or (clinimetr* or clinometr*).tw. or exp "Outcome Assessment (Health Care)"/ or outcome assessment.ti,ab. or outcome measure*.tw. or exp Observer Variation/ or observer variation.ti,ab. or exp Health Status Indicators/ or exp "Reproducibility of Results"/ or reproducib*.ti,ab. or exp Discriminant Analysis/ or (reliab* or unreliab* or valid* or coefficient or homogeneity or homogeneous or "internal consistency").ti,ab. or (cronbach* and (alpha or alphas)).ti,ab. or (item and (correlation* or selection* or reduction*)).ti,ab. or (agreement or precision or imprecision or "precise values" or test-retest).ti,ab. or (test and retest).ti,ab. or (reliab* and (test or retest)).ti,ab. or (stability or interrater or inter-rater or intrarater or intra-rater or intertester or inter-tester or intratester or intra-tester or interobserver or inter-observer or intraobserver or intraobserver or intertechnician or inter-technician or intratechnician or intra-technician or interexaminer or inter-examiner or intraexaminer or intra-examiner or interassay or interassay or intraassay or intra-assay or interindividual or inter-individual or intraindividual or intra-individual or interparticipant or inter-participant or intraparticipant or intra-participant or kappa or kappa's or kappas or repeatab*).ti,ab. or ((replicab* or repeated) and (measure or measures or findings or result or results or test or tests)).ti,ab. or (generaliza* or generalisa* or concordance).ti,ab. or (intraclass and correlation*).ti,ab. or (discriminative or "known group" or factor analysis or factor analyses or dimension* or subscale*).ti,ab. or (multitrait and scaling and (analysis or analyses)).ti,ab. or (item discriminant or interscale correlation* or error or errors or "individual variability").ti,ab. or (variability and (analysis or values)).ti,ab. or (uncertainty and (measurement or measuring)).ti,ab. or ("standard error of measurement" or sensitiv* or responsive*).ti,ab. or ((minimal or minimally or clinical or clinically) and (important or significant or detectable) and (change or difference)).ti,ab. or (small* and (real or detectable) and (change or difference)).ti,ab. or (meaningful change or "ceiling effect" or "floor effect" or "Item response model" or IRT or Rasch or "Differential item functioning" or DIF or "computer adaptive testing" or "item bank" or "cross-cultural equivalence").ti,ab. (12549272)

5 (addresses or biography or case reports or comment or directory or editorial or festschrift or interview or lectures or legal cases or legislation or letter or news or newspaper article or patient education handout or popular works or congresses or consensus development conference or consensus development conference, nih or practice guideline).pt. not (*animals/ not *humans/) (5263211)

6 (1 and 2 and 3 and 4) not 5 (347)

7 limit 6 to english language (341)

8 limit 7 to human (316)

9 limit 8 to yr="1980 -Current" (316)

***************************

Search 2 – Named measures search

Database: Ovid MEDLINE(R) <1946 to August Week 5 2017>, Embase Classic+Embase <1947 to 2017 September 08>, PsycINFO <1967 to September Week 1 2017>

Search Strategy:

--------------------------------------------------------------------------------

1 Spondylitis, Ankylosing/ or (Ankylosing Spondyl* or Spondyl*, Ankylosing or Spondyloarthropath* or Spondyloarthritis).ti,ab. (49014)

2 (Fatigue or Asthenia* or Lassitude or Exhaust* or Inertia or Drows* or Letharg* or (Tiring or Tired* or Weary or Weariness)).mp. (544147)

3 (Fatigue Assessment Scale or FAS or Fatigue Impact Scale or FIS or Fatigue Scale or FS or Fatigue Symptom Inventory or FSI or Myasthenia Gravis Fatigue Scale or Multidimensional Fatigue Symptom Inventory or Multi-dimensional Fatigue Symptom Inventory or MFSI or MFSI-SF or Parkinsons Fatigue Scale or (Pearson and Byars Fatigue Feeling Checklist) or Revised Piper Fatigue Scale or Piper Fatigue Scale or R-PFS or PFS or Rhoten Fatigue Scale or (Schedule of Fatigue and Anergia) or SOFA or Schwartz Cancer Fatigue Scale or SCFS or Visual Analog Fatigue Scale or VAS-F or Checklist Individual Strength or CIS20R or CIS8R or Multidimensional Fatigue Scale or Multi-dimensional Fatigue Scale or MFS or Fatigue Questionnaire or Multidimensional Assessment of Fatigue or Multi-Dimensional Assessment of Fatigue or MAF or Multi-dimensional Health Assessment Questionnaire or Multidimensional Health Assessment Questionnaire or MDHAQ or Profile of Fatigue or ProF or Multidimensional Fatigue Inventory or Multi-dimensional Fatigue Inventory or MFI or Bath Ankylosing Spondylitis Disease Activity Index or BASDAI or (mini adj BASDAI) or ((Bristol Rheumatoid Arthritis Fatigue Multidimensional Questionnaire adj MDQ) or NRS) or ((Bristol Rheumatoid Arthritis Fatigue Multi-dimensional Fatigue Questionnaire adj MDQ) or NRS) or ((BRAF adj MDQ) or NRS) or BRAFMDQ or BRAFNRS or Short-Form Health Survey or SF-36 or SF36 or SF36-V2 or SF36V2 or Bath Ankylosing Spondylitis Functional Index or BASFI or Evaluation of Ankylosing Spondylitis Quality of Life or EASi-QoL or EASiQoL or Brief Fatigue Inventory or BFI or Functional Assessment of Chronic Illness Therapy or (FACIT adj (F or Fatigue)) or Evaluation of Daily Activity Questionnaire or EDAQ or Worst Fatigue-Numeric Rating Scale or WF-NRS or WFNRS or Fatigue Severity Scale or FSS or Chalder Fatigue Scale or CFS or Patient Reported Outcome* Measurement Information System or PROMIS Visual Analogue Scale* or VAS or Numeric Rating Scale* or NRS).mp. (436787)

4 (instrumentation or methods).sh. or (Validation Studies or Comparative Study).pt. or exp Psychometrics/ or psychometr*.ti,ab. or (clinimetr* or clinometr*).tw. or exp "Outcome Assessment (Health Care)"/ or outcome assessment.ti,ab. or outcome measure*.tw. or exp Observer Variation/ or observer variation.ti,ab. or exp Health Status Indicators/ or exp "Reproducibility of Results"/ or reproducib*.ti,ab. or exp Discriminant Analysis/ or (reliab* or unreliab* or valid* or coefficient or homogeneity or homogeneous or "internal consistency").ti,ab. or (cronbach* and (alpha or alphas)).ti,ab. or (item and (correlation* or selection* or reduction*)).ti,ab. or (agreement or precision or imprecision or "precise values" or test-retest).ti,ab. or (test and retest).ti,ab. or (reliab* and (test or retest)).ti,ab. or (stability or interrater or inter-rater or intrarater or intra-rater or intertester or inter-tester or intratester or intra-tester or interobserver or inter-observer or intraobserver or intraobserver or intertechnician or inter-technician or intratechnician or intra-technician or interexaminer or inter-examiner or intraexaminer or intra-examiner or interassay or interassay or intraassay or intra-assay or interindividual or inter-individual or intraindividual or intra-individual or interparticipant or inter-participant or intraparticipant or intra-participant or kappa or kappa's or kappas or repeatab*).ti,ab. or ((replicab* or repeated) and (measure or measures or findings or result or results or test or tests)).ti,ab. or (generaliza* or generalisa* or concordance).ti,ab. or (intraclass and correlation*).ti,ab. or (discriminative or "known group" or factor analysis or factor analyses or dimension* or subscale*).ti,ab. or (multitrait and scaling and (analysis or analyses)).ti,ab. or (item discriminant or interscale correlation* or error or errors or "individual variability").ti,ab. or (variability and (analysis or values)).ti,ab. or (uncertainty and (measurement or measuring)).ti,ab. or ("standard error of measurement" or sensitiv* or responsive*).ti,ab. or ((minimal or minimally or clinical or clinically) and (important or significant or detectable) and (change or difference)).ti,ab. or (small* and (real or detectable) and (change or difference)).ti,ab. or (meaningful change or "ceiling effect" or "floor effect" or "Item response model" or IRT or Rasch or "Differential item functioning" or DIF or "computer adaptive testing" or "item bank" or "cross-cultural equivalence").ti,ab. (12549272)

5 1 and 2 and 3 and 4 (319)

6 limit 5 to english language (312)

7 limit 6 to human (288)

8 limit 7 to yr="1980 -Current" (288)

9 remove duplicates from 8 (220)

***************************

**Appendix 2: Quality criteria to appraise reported measurement properties [29,36]**

| **Measurement properties** | **Rating** | **Quality criteria** |
| --- | --- | --- |
| **Validity** |  |  |
| Content validity | + | Authors provide a clear description of the measurement aim, target population, concept(s) measured and process of item selection.  Members of the target population and experts in the field were clearly identified as being involved in development. For measures applied  for the first time in a new population, evidence that the views of members of the target population (and experts in the field) have been  sought to determine relevance, comprehension and comprehensiveness. |
|  | ? | Insufficient evidence available |
|  | - | No detail re measurement aim, target population, concept(s) measured, process of item selection; members of the target population or  experts were not specifically involved in development.  For measures applied for the first time in a new population, evidence whereby the relevance and acceptability of the measure with  members of the target audience or experts was not provided. |
|  |  |  |
| Construct validity – Structural validity | + | Factors should explain at least 50% of the variance |
|  | ? | Explained variance not stated |
|  | - | Factors explain <50% of the variance |
|  |  |  |
| Construct validity – Hypothesis testing | + | Correlations with measures of the same construct should be >0.50 OR at least 75% of the results in accordance with hypothesized  associations AND correlations with related constructs should be higher than with those reported with unrelated constructs |
|  | ? | Only report correlations with unrelated constructs OR the extent to which between group differences are expected is not described /  justified |
|  | - | Correlations with measures of the same construct are <0.50 OR < 75% of the results in accordance with hypothesized associations OR  correlations with related constructs are lower than those reported with unrelated constructs |
|  |  |  |
| Construct validity – Known-group validity  (not part of the COSMIN checklist) | + | Hypothesised between group differences are supported (or can be assumed) AND between group differences are statistically significant |
|  | ? | Between group differences are poorly hypothesized, but between group differences are statistically significant |
|  | - | Expected between group difference poorly defined or justified AND the statistical significance of between group differences not reported |
|  |  |  |
| **Reliability** |  |  |
| Internal consistency | + | Cronbach’s alpha(s) ≥ 0.70 |
|  | ? | Cronbach’s alpha not determined or dimensionality unknown |
|  | - | Cronbach’s alpha(as) < 0.70 |
|  |  |  |
| Reliability (test-retest / inter-rater / intra-rater) | + | Intra-class Correlation Coefficient (ICC)/ weighted Kappa ≥0.70 OR Pearson’s r ≥0.80 |
|  | ? | Neither ICC/ weighted Kappa, nor Pearson’s r established |
|  | - | ICC/ weighted Kappa <0.70 OR Pearson’s r <0.80 |
|  |  |  |
| Reliability – measurement error | N/A | Descriptive (not rated) |
|  |  |  |
| Responsiveness | + | Change-score correlations with measures of the same construct are >0.50 OR at least 75% of the results are in accordance with  hypothesized associations OR the Area Under the Curve (AUC) is >0.70 AND change-score correlations with measures of related constructs  are higher than those reported with unrelated constructs |
|  | ? | Solely correlations with unrelated constructs |
|  | - | Change-score correlations with measure of the same construct <0.50 OR < 75% of the results are in accordance with hypothesized  associations OR AUC is <0.70 AND change-score correlations with related constructs are lower than those reported with unrelated  constructs |
|  |  |  |
| Interpretability | N/A | Descriptive (not rated) - requires evidence that the minimal important (within-person) change (MIC) and/or minimal importance (between  group) difference (MID) exceeds evidence of the smallest detectable difference (SDD). Supported by evidence of acceptable data quality  (score distribution, absence of end effects (floor/ ceiling) |
